# Screening of key genes in the pathogenesis of muscle atrophy in CKD-PEW children based on RNA sequencing

**DOI:** 10.1186/s12920-023-01718-1

**Published:** 2023-11-28

**Authors:** Liang Ying, Jiang Yeping, Wang Hui, Zhou Nan, FuQian, Shen Ying

**Affiliations:** Department 2 of Nephrology, Beijing Key Laboratory for Chronic Renal Disease and Blood Purification, Beijing Children′s Hospital Affiliated to Capital Medical University, Key Laboratory of Major Diseases in Children, National Center for Children’s Health, China, Beijing, 100045 China

**Keywords:** Chronic kidney disease, Protein-energy wasting, Muscle atrophy, Key genes, NF-κB, RNA sequencing

## Abstract

**Background:**

In children with CKD, Protein Energy Wasting (PEW) is common, which affects the outcome of children and is an important cause of poor prognosis. We are aiming to explore the pathogenesis of muscle wasting in CKD-PEW children.

**Methods:**

Blood samples of 32 children diagnosed with chronic kidney disease (CKD) and protein energy wasting (PEW) in our hospital from January 2016 to June 2021 were collected. RNA sequencing and bioinformatics analysis were performed.

**Results:**

Based on GO (Gene Ontology) functional enrichment analysis, KEGG (Kyoto Encyclopedia of Genes and Genomes) pathway enrichment analysis and differential gene expression analysis, a total of 25 CKD-PEW related genes were obtained including CRP, IL6, TNF, IL1B, CXCL8, IL12B, IL12A, IL18, IL1A, IL4, IL10, TGFB2, TGFB1, TGFB3, ADIPOQ, NAMPT, RETN, RETNLB, LEP, CD163, ICAM1, VCAM1, SELE, NF-κB1, NF-κB2. The most significantly differentially expressed gene was NF-κB2 (adjusted *P* = 2.81 × 10^–16^), and its expression was up-regulated by 3.92 times (corresponding log2FoldChange value was 1.979). Followed by RETN (adjusted *P* = 1.63 × 10^–7^), and its expression was up-regulated by 8.306 times (corresponding log2FoldChange value was 2.882). SELE gene were secondly significant (adjusted *P* = 5.81 × 10^–7^), and its expression was down-regulated by 22.05 times (corresponding log2FoldChange value was -4.696).

**Conclusions:**

A variety of inflammatory factors are involved in the pathogenesis of CKD-PEW in children, and chronic inflammation may lead to the development of muscle atrophy in CKD-PEW. It is suggested for the first time that NF-κB is a key gene in the pathogenesis of muscle wasting in CKD-PEW children, and its increased expression may play an important role in the pathogenesis of muscle wasting in children with CKD-PEW.

## Introduction

Chronic Kidney Disease (CKD) involves a gradual loss of kidney function. Malnutrition is an important complication of CKD and leads to poor prognosis [1], but there has always been a lack of quantifiable indicators to evaluate malnutrition. In 2006, the International Society for Renal Nutrition and Metabolism (ISRNM) proposed the concept of protein energy expenditure (PEW) [2], which serves as a quantifiable diagnostic standard for describing malnutrition. PEW refers to loss of body protein mass and fuel reserves. In 2014, Abraham et al. conducted a study on 528 children with CKD based on the four evaluation aspects of adult PEW diagnostic criteria. Combining the characteristics of children, they added the fifth growth and development indicator evaluation. Using these five diagnostic criteria, Abraham et al. proposed three different diagnostic recommendations that can be used for evaluating pediatric CKD PEW. According to different diagnostic conditions, they were divided into mild PEW, standard PEW, and modified PEW. Mild PEW refers to a child who meets any two of the first four diagnostic criteria; Standard PEW refers to a child who meets any three of the first four diagnostic criteria; Revised PEW refers to meeting any three of the five diagnostic criteria and must include developmental delay [3]. PEW can lead to an increase in the mortality rate of CKD and induce the progress to end-stage renal disease (ESRD),which brings heavy economic burden to families and society. PEW is very common in children with CKD. And PEW is an independent risk factor for poor prognosis of CKD [4].

Transcriptome refers to the sum of all RNAs transcribed in a specific tissue or cell at a certain time or state, mainly including mRNA and non-coding RNA [5]. RNA sequencing is based on Illumina sequencing platform to study all mRNA transcribed in a specific tissue or cell at a definite period [6]. It is the basis of gene function and structure research, and plays a major role in understanding the development of organisms and the occurrence of diseases. With the development of gene sequencing technology and the reduction of sequencing cost, RNA sequencing has become the main method for transcriptome research due to its advantages of high throughput, high sensitivity and wide application range. The RNA sequencing technical process mainly consists of two parts: database construction and bioinformatics analysis.

The pathogenesis of PEW in children with CKD is very complex, and the exact mechanism is currently unclear. Some studies have shown that it is related to factors such as decreased appetite, inflammatory state, insufficient energy intake, increased consumption, and dialysis, etc. [7]. However, research on children in this related field is almost blank. We plan to start by exploring the key genes involved in the pathogenesis of PEW in children with CKD based on RNA sequencing, which will help to understand the process of the occurrence and development of PEW, and furtherly provide basis for clinical intervention and treatment to improve the prognosis of CKD- PEW patients.

## Materials and methods

A total of 32 children diagnosed with CKD PEW in our hospital from January 2016 to June 2021 were enrolled. The study was approved by the Ethics committee of Beijing Children's Hospital and strictly followed the Declaration of Helsinki.

### Inclusion criteria

(1) Diagnostic criteria for chronic kidney disease [8]: ① Kidney injury (including abnormal urine test, imaging or pathological examination) ≥ 3 months; ② Estimated glomerular filtration rate (eGFR) < 60 ml/(min·1.73 m^2^) ≥ 3 months, with or without evidence of kidney injury.

According to different severity levels, CKD can be divided into 5 stages based on eGFR, with later stages becoming more severe:

- Stage 1 eGFR ≥ 90 ml/(min · 1.73 m^2^);
- Stage 2 eGFR 60–89 ml/(min · 1.73 m^2^);
- Stage 3 eGFR 30–59 ml/(min · 1.73 m^2^);
- Stage 4 eGFR 15–29 ml/(min · 1.73 m^2^);
- Stage 5 eGFR < 15 ml/(min · 1.73 m^2^).

(2)The diagnostic criteria for protein-energy wasting were based on the adult diagnostic criteria of the International Society of Renal Nutrition and Metabolism, and the modified diagnostic criteria for children were combined with the characteristics of children and relevant literature, including [3, 9, 10]: (1) Biochemical indicators: albumin < 38 g/L, prealbumin < 300 mg/L, total cholesterol < 100 mg/100 mL, serum transferrin < 140 mg/dl, CRP > 3 mg/L; (2) body weight: BMI below the 5th percentile of the same age and sex or BMI below the 80th percentile of the same age and sex but BMI decreased by ≥ 10% in one year; (3) Low muscle mass: mid-upper arm circumference (MUAC) below the 5th percentile of the same age and sex or MUAC decline ≥ 10% within one year; (4) insufficient diet: decreased appetite, poor or very poor appetite in the past week, insufficient protein intake; (5) Growth retardation: short stature (height below the 3rd percentile of the same age and sex or slow growth rate (annual growth rate is more than 10% behind the population of the same age and sex). PEW definition requiring any positive test in ≥ 3 of categories (1) through (5), such that poor growth was also included as a separate indicator category.

### Exclusion criteria

(1) complicated with other organ dysfunction; (2) complicated with infection; (3) receiving blood purification treatment.

### Methods

Three to four ml blood samples were drawn from each child and stored in PAXgene Blood RNA storage tubes of BD company. Blood samples were kept with 4° ice bags, placed in an incubator, and delivered immediately for RNA sequencing. RNA-seq experiment and high through-put sequencing and data analysis were conducted by MyGenostics Inc. (Beijing, China).

#### RNA extraction, library preparation and sequencing

Total RNAs were extracted from peripheral blood cells of 32 children with CKD PEW using TRIzol Reagent (Invitrogen, cat. NO 15596026) following the methods by Chomczynski et al. (https://doi.org/10.1006/abio.1987.9999). DNA digestion was carried out after RNA extraction by DNaseI. RNA quality was determined by examining A260/A280 with NanodropTM OneCspectrophotometer (Thermo Fisher Scientific Inc). RNA Integrity was confirmed by 1.5% agarose gel electrophoresis. Qualified RNAs were finally quantified by Qubit3.0 with QubitTM RNA Broad Range Assay kit (Life Technologies, Q10210).

Two μg total RNAs were used for stranded RNA sequencing library preparation using KCTM Stranded mRNA Library Prep Kit for Illumina® (Catalog NO. DR08402, Wuhan Seqhealth Co., Ltd. China) following the manufacturer’s instruction. PCR products corresponding to 200–700 bps were enriched, quantified and finally sequenced on DNBSEQ-T7 sequencer (MGI Tech Co., Ltd. China) with PE150 model.

**Fig. 1:**
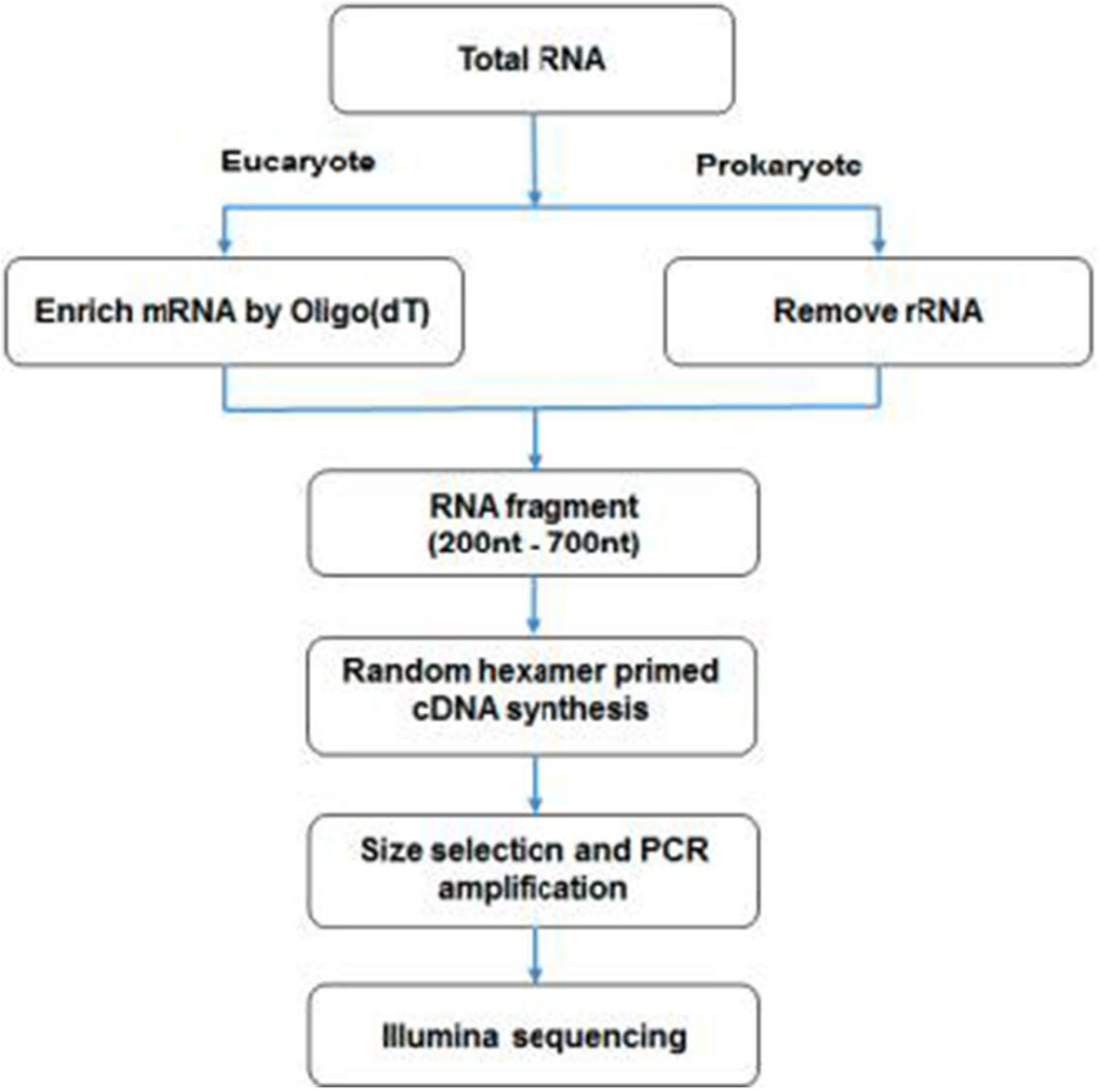
Transcriptome experiment process

In this study, the eukaryotic mRNA was enriched by magnetic beads with Oligo (dT). The mRNA was broken into short fragments by adding lysis buffer. The first cDNA strand was synthesized with random hexamers using mRNA as template, and then the second cDNA strand was synthesized by adding buffer, dNTPs, RNase H and DNA polymerase I. After purification by QiaQuick PCR kit, elution with EB buffer and the end repair, polyA and sequencing adapter were connected, and then agarose gel electrophoresis was used for fragment size selection, and finally PCR amplification was performed. The constructed sequencing library was sequenced by high-throughput sequencing instrument. The specific experimental procedure is shown in Fig. 1.

#### RNA-Seq data analysis

Raw sequencing data was first filtered by Trimmomatic (version 0.36), low-quality reads were discarded and the reads contaminated with adaptor sequences were trimmed.clean data were mapped to the reference genome of human from Ensembl database (ftp://ftp.ensembl.org/pub/release-87/fasta/homo_sapiens/dna/) using STRA software (version 2.5.3a) with default parameters. Reads mapped to the exon regions of each gene were counted by featureCounts (Subread-1.5.1; Bioconductor) and then RPKMs were calculated. Genes differentially expressed between groups were identified using the edgeR package (version 3.12.1). A *p*-value cutoff of 0.05 and fold-change cutoff of 2 were used to judge the statistical significance of gene expression differences. Gene ontology (GO) analysis and Kyoto encyclopedia of genes and genomes (KEGG) enrichment analysis for differentially expressed genes were both implemented by KOBAS software (version: 2.1.1) with a *p* value cutoff of 0.05 to judge statistically significant enrichment. Alternative splicing events were detected by using rMATS (version 3.2.5) with a FDR value cutoff of 0.05 and an absolute value of Δψ of 0.05.

**Fig. 2:**
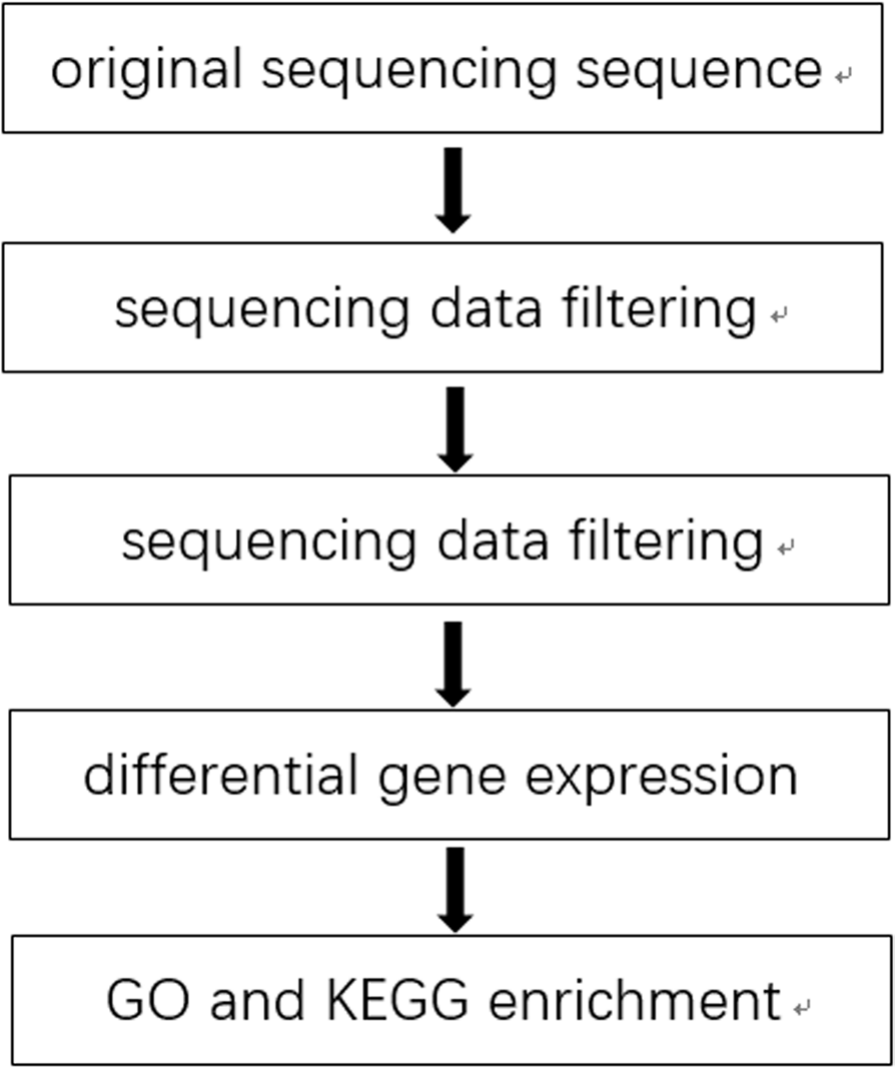
Biological information analysis process

Once Sequenced Reads have been obtained and their corresponding Reference Genome sequences are available, the data can be analyzed in detail using a reference genome information analysis procedure. Bioinformatics analysis method was used to select significant differentially expressed genes (DEGs), and cluster analysis, gene ontology (GO) functional annotation analysis, Kyoto Encyclopedia of Genes and Genomes (KEGG) pathway enrichment analysis were performed for significant DEGs. The analysis process is shown in Fig. 2.

### Statistical method

SPSS19.0 software was used to establish a database and analyze the data. *P* < 0.05 was considered statistically significant.

## Results

A total of 32 children were included in the study, with an average age of 9.27 years, including 21 males and 11 females.

### Differential gene analysis

DESeq2 software was used to analyze 32 cases versus 18 controls [11]. A total of 57,773 expression profiles were generated, and 18,796 genes were significantly differentially expressed (*P* < 0.05), of which 4998 genes were up-regulated, 13,798 genes were down-regulated. There were 1102 genes with log2foldchange > 2 and 10,495 genes with log2foldchange < -2.

### GO functional annotation analysis of differential genes

**Fig. 3:**
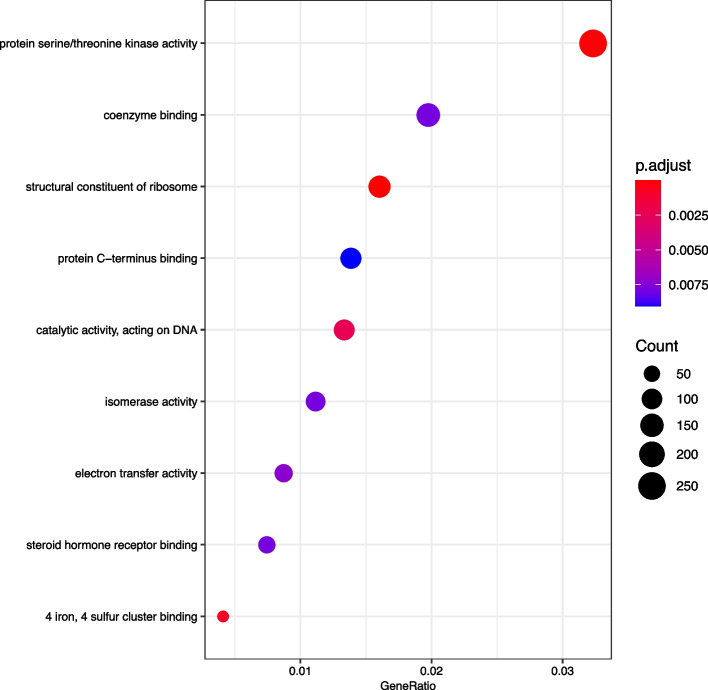
GO function annotation analysis

GO functional annotation analysis found the top 9 GO terms with the most obvious enrichment (see Fig. 3).

### KEGG pathway enrichment analysis

**Fig. 4:**
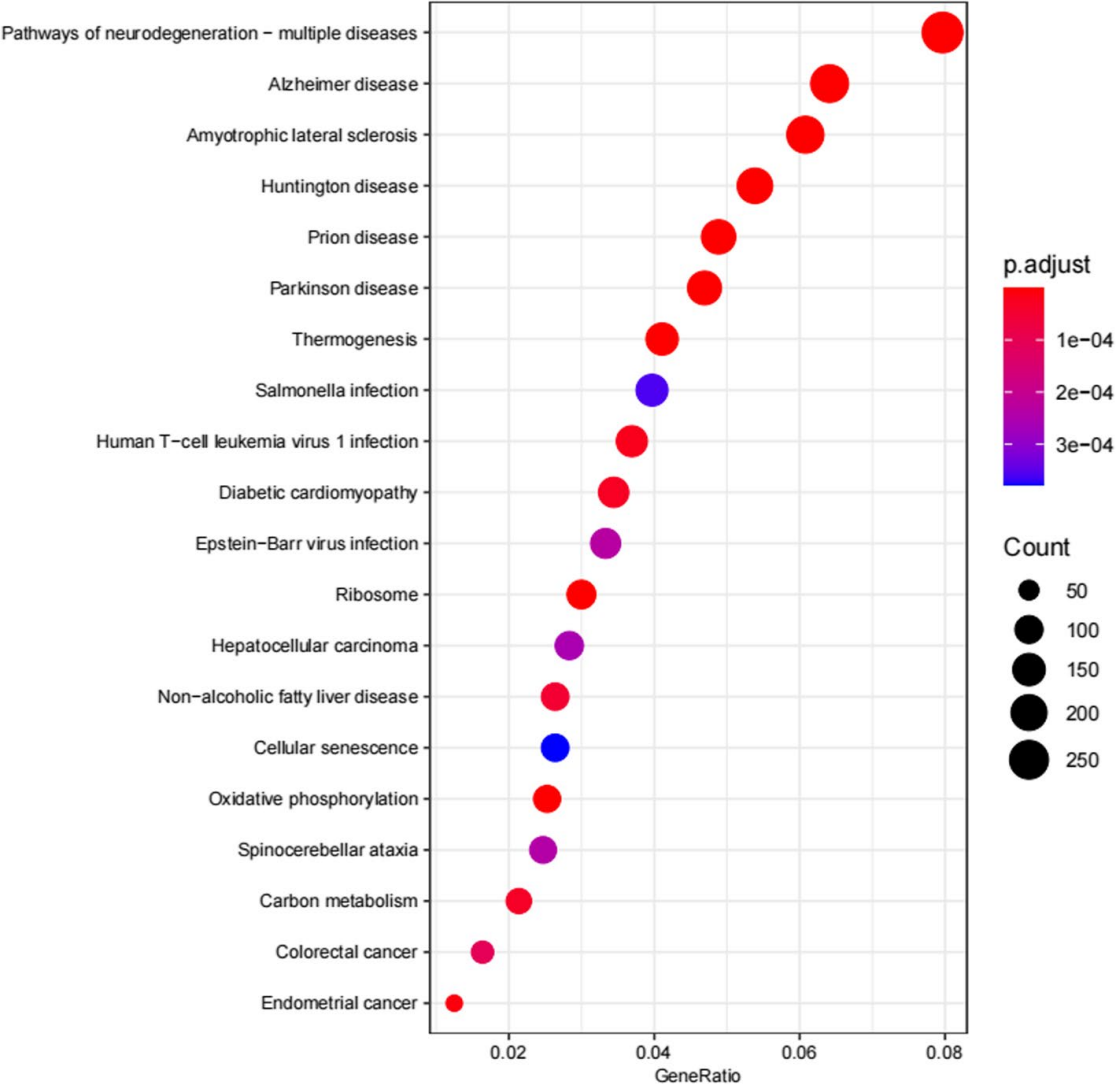
Enrichment analysis results of KEGG pathway

KEGG pathway enrichment analysis found the top 20 most significantly enriched related pathways (see Fig. 4). pathways are included in Table 1.

**Table 1:** Signaling pathways involved after screening

ID	Description	Gene Ratio	Bg Ratio	*p* value	p.adjust	q value	Count
hsa 05022	Pathways of neurodegeneration-multiple diseases	287/3603	475/8102	4.63E-13	6.61E-11	4.84E-11	287
hsa 04066	H IF-1signalng pathway	67/3603	109/8102	0.000242821	0.003063286	0.002241429	67
hsa 04662	Bc ll receptor signaling pathway	52/3603	82/8102	0.00040248	0.003945969	0.002887294	52
hsa 04919	Thyroid hormone signaling pathway	72/3603	121/8102	0.000576795	0.005266009	0.003853177	72
hsa 05235	PD-L 1 expression and PD-1 checkpoint pathway in cancer	55/3603	89/8102	0.0007067	0.006264796	0.004583997	55
hsa 04722	Neurotrophin signaling pathway	69/3603	119/8102	0.001954049	0.015260194	0.011165996	69
hsa 03460	Fanconi anemia pathway	34/3603	54/8102	0.004654828	0.027263995	0.019949265	34
hsa 04370	VEGF signaling pathway	36/3603	59/8102	0.007576716	0.040083273	0.029329224	36
hsa 04115	p 53 signaling pathway	43/3603	73/8102	0.008963409	0.043880568	0.032107733	43
hsa 04910	Insulin signaling pathway	75/3603	137/8102	0.009480865	0.045731233	0.033461878	75

After enrichment results of clearly related diseases are removed, the signal pathways clearly involved are included as shown in Table 1.

### Differentially expressed gene analysis of related genes

In this study, genes associated with CKD-PEW for which expression profile data were obtained included: CRP, IL6, TNF, IL1B, CXCL8, IL12B, IL12A, IL18, IL1A, IL4, IL10, TGFB2, TGFB1, TGFB3, ADIPOQ, NAMPT, RETN, RETNLB, LEP, CD163, ICAM1, VCAM1, SELE, NF-κB1, NF-κB2, a total of 25 genes (Table 2). The most significantly differentially expressed gene was NF-κB2 (adjusted *P* = 2.81 × 10^–16^), and its expression was up-regulated by 3.92 times (corresponding log2FoldChange value was 1.979). Followed by RETN (adjusted *P* = 1.63 × 10^–7^), and its expression was up-regulated by 8.306 times (corresponding log2FoldChange value was 2.882). SELE gene was secondly significant (adjusted *P* = 5.81 × 10^–7^), and its expression was down-regulated by 22.05 times (corresponding log2FoldChange value was -4.696).

**Table 2:** Differential expression of PEW-related genes

Symbol	base Mean	log2Fold Change	lfcS E	stat	*p* value	pad j
CRP	0.114060697	-2.465777735	3.052606957	-0.80776129	0.419228013	NA
IL6	4.337756836	-2.097894186	0.668834639	-3.136641051	0.001708952	0.004291322
TNF	24.24167726	0.204278682	0.320119511	0.638132558	0.523387395	0.586191744
IL1B	128.893596	0.771694378	0.533840313	1.445552834	0.148302664	0.219156753
IL12B	1.808449852	-2.877527179	0.969963121	-2.966635656	0.003010775	0.007193719
IL12A	1.080084815	-0.055874831	0.765793921	-0.072963273	0.941835344	0.952752572
IL18	18.99090481	0.465493318	0.374592386	1.242666255	0.213990844	0.296892669
IL1A	1.481022278	-3.353496342	1.456555639	-2.302346887	0.021315618	0.04171952
IL4	3.984502138	-2.925117084	0.916738286	-3.190787523	0.001418856	0.003614539
IL10	2.047947257	0.18102241	0.798008773	0.226842631	0.820546113	0.848928891
TGFB2	2.018770219	-0.446821797	1.173327605	-0.380815891	0.703339867	0.747362496
TGFB1	3214.310804	-0.961412983	0.272018251	-3.534369397	0.000408749	0.001145215
TGFB3	13.00439431	-0.517424559	0.244181328	-2.119017712	0.03408897	0.062938339
ADIPOQ	0.830878186	-2.023824739	2.597678203	-0.779089857	0.435926785	0.515006899
NAM PT	2566.730162	0.372143322	0.418623439	0.888969146	0.374019662	0.461707141
RETN	36.73594024	2.881988542	0.522733974	5.513298708	3.52E-08	1.63E-07
RETNLB	0	0	0	0	1	NA
LEP	2.499951577	0.232589355	0.757807648	0.306924001	0.75890122	0.795951145
CD163	115.8437443	0.181758807	0.490659937	0.370437432	0.711056588	0.754490044
ICAM 1	123.500927	0.934721729	0.39308785	2.377895247	0.017411771	0.034945658
VCAM 1	0.572781918	-1.403888981	0.883067187	-1.589787279	0.111882762	0.173397464
SELE	2.930089268	-4.696032052	0.890352922	-5.274349009	1.33E-07	5.81E-07
NFKB 1	267.9243891	-0.087666353	0.156330486	-0.560775801	0.574950385	0.631769508
NFKB 2	657.4549521	1.97914639	0.234175044	8.451568355	2.87E-17	2.81E-16
CXC L 8	35.15898113	-1.515208513	0.674984538	-2.244804772	0.024780668	0.047642656

*Symbol* gene name, *baseMean* readcount average, *log2FoldChange* log2(group1 baseMean/group2 baseMean), *lfcSe* log2FoldChange standard deviation, *p value* statistical significance test index, *padj* corrected *p* value

## Discussion

Chronic Kidney Disease (CKD) is a worldwide epidemic disease. In children with CKD, Protein Energy Wasting (PEW) is common, which affects the outcome of children and is an important cause of poor prognosis [12, 13]. Research on the etiology and pathogenesis of malnutrition in chronic kidney disease involves many factors such as decreased appetite, micro-inflammatory state, insufficient energy intake, increased energy consumption, and dialysis. Inflammation is one of the principal factors leading to PEW, especially muscle atrophy [14, 15].

### CKD PEW is closely related to inflammation.

Inflammation is an important component of chronic kidney disease (CKD), which has been recognized since the late 1990s and is associated with PEW and mortality [16]. In the past two decades, research on CKD and inflammation has been increasing. When the body is stimulated by microorganisms, endotoxins, chemicals, immune complexes, complement, etc., the inflammatory response occurs with the release of pro-inflammatory factors at the center [17]. Pro-inflammatory factors including tumor necrosis factor-α (TNF-α), interleukin-1 (IL-1), interleukin-6 (IL-6), interferon ɣ (IFN-ɣ) activate nuclear factor kappa b(NF-κB), Activation of the Ubiquitin proteasome system (UPS) induces increased proteolysis and muscle protein breakdown. In addition, inflammation can also inhibit protein synthesis [18]. In addition, inflammation can also inhibit protein consumption by inhibiting the insulin-like growth factor 1(IGF1)/phosphatidylinositol kinase (PI3K)/protein kinase B(Akt) signaling pathway. With the increasing understanding of protein wasting, some scholars have noted that increased protein breakdown plays a dominant role in CKD-related PEW. It has been reported that 80%-90% of cellular proteins are degraded through the ubiquitin proteasome system, which is considered to be the most important protein degradation system in cells. The ubiquitin proteasome system includes ubiquitin, ubiquitin activating enzyme (E1), ubiquitin coupling enzyme (E2), ubiquitin-protein ligase (E3), and the proteasome. The main function of ubiquitin is to covalently bind to misfolded or short-lived proteins in the cytoplasm. The ubiquitin proteasome system degrades the target protein into peptide fragments by binding ubiquitin to the substrate protein and then recognizing the protein to be degraded.E1 first activates ubiquitin, E2 binds to activated ubiquitin and transports it to target proteins, and E3 regulates E2 so that it accurately transports ubiquitin to target proteins. While more than 500 E3s have been identified, two specific E3s have been identified for PEW: muscle-specific ring finger protein (MuRF1) and muscle atrophy cassette protein (MAFbx, otherwise known as Atrogin). They were all elevated several-fold in uremic mice, whereas MuRF1 and MAFbx were barely expressed during normal muscle growth.These two proteins are currently considered to be markers of proteolysis and muscle atrophy. The 26S proteasome, consisting of a 20S core proteasome and two 19S regulatory complexes, is the key enzyme that catalyzes the degradation of the ubiquitin-substrate protein coupling. The process of the ubiquitin proteasome system is as follows: first, with the participation of ATP, ubiquitin activates E1, E2, and E3 to form the ubiquitin-protein coupling. The coupling is then recognized by the 26S proteasome, resulting in its degradation, and ubiquitin returns to the cycle to participate in protein degradation. In this study, differential gene analysis of 32 children with CKD-PEW concluded that the related genes included: CRP, IL6, TNF, IL1B, CXCL8, IL12B, IL12A, IL18, IL1A, IL4, IL10, TGFB2, TGFB1, TGFB3, ADIPOQ, NAMPT, RETN, RETNLB, LEP, CD163, ICAM1, VCAM1, SELE, NFKB1, NF-ΚB and so on, a total of 25 genes, including CRP, IL6, TNF, IL1B, IL12B, IL12A, IL18, IL1A, IL4, IL10, NFKB1, NF-ΚB and many other genes have been reported to be closely related to chronic inflammatory response, which is consistent with the our conclusion [19].

### NF-κB promotes muscle atrophy by mediating inflammation

The current mainstream view is that CKD-induced muscle atrophy is closely related to inflammation. In chronic diseases, inflammatory factors can activate different cellular signals upon binding to their receptors, including camp-dependent protein kinases (PKA), protein kinase C (PKC), phosphatidylinositol 3-kinases (PI3-K), and mitogen-activated protein kinases (MAPKs).The stimulation of these proteins kinases leads to the activation of different transcription factors, of which NF-κB is one of the important signaling pathways [20, 21]. NF-κB, a key nuclear factor that regulates genes in a variety of chronic inflammatory diseases, has been shown to play an important role in diverse disease-related muscular atrophy. In activating muscle atrophy gene expression, NF-κB is one of the key regulator mediating inflammatory responses and apoptosis, assisting FOXO transcription factors to exert their effects. It has been demonstrated that inhibition of NF-κB expression in muscle by IκB, an inhibitor of NF-κB, can slow down denervated muscle atrophy. In mice lacking IκB kinase-β, IκB degradation is reduced. NF-κB activation is reduced, and denervated muscle atrophy can be alleviated. The various upstream signals that regulate NF-κB function in muscle in different disease-associated muscular atrophy have not meant fully identified. However, it has been shown that TNF-α, IL-6, IL-1 and IFN-γ are elevated in sepsis, cancer and other catabolic states, and may jointly cause muscle atrophy by increasing the expression of NF-κB. Although it has been reported that NF-κb has a potent induction effect on Myostatin, the exact mechanism by which NF-κB promotes muscle atrophy is still unclear. Cohen et al. suggested that NF-κB activation activates some components of the ubiquitin-protease system, such as MuRF1 and MAFbx, and the activation of the ubiquitin protease system promotes the degradation of muscle proteins; In turn, NF-κB exerts a positive feedback effect on the expression of pro-inflammatory factors, increasing the protein level of pro-inflammatory factors that it regulates. This mechanism overactivaty NF-κB and subsequently induces muscle atrophy. The results of the present study showed that the transcriptional level of NF-κB gene in 32 CKD patients was significantly higher than that in controls, and the up-regulation ratio was 3.92 times, which was also consistent with the above study. The increased expression of NF-κB may lead to muscle atrophy in children with protein-energy wasting in CKD.

### More evidence is needed to confirm the involvement of other genes in muscle atrophy of CKD-PEW

The transcription levels of RETN and SELE genes in patients with CKD were also significantly higher than those in the control group. Because the proportion of up-regulation was the same, it is speculated that RETN and SELE genes may play a synergistic role. KEGG only found that SELE was involved in the "Fluid shear stress and atherosclerosis" signaling pathway, but RETN was not found to be involved in the signaling pathway. According to the literature search, only one article showed that RETN and SELE polymorphisms were significantly associated with coronary artery disease, but the relationship between RETN and SELE polymorphisms and CKD was not reported (PMID: 24,699,314).Further studies are needed to explore the association between Retn and SELE polymorphisms and CKD-PEW.

Inflammation is one of the important factors leading to protein-energy wasting in chronic renal failure, especially muscle wasting [22]. NF-κB is a key nuclear factor that regulates genes in a variety of chronic inflammatory diseases. There have been few studies of the mechanism of CKD-PEW, especially in children. This study takes the lead in finding that NF-κB signaling pathway may play a key role in the pathogenesis of CKD-PEW muscle atrophy in children. We are conducting relevant animal experiments in mice with chronic renal failure to further verify and explore the role of NF-κB signaling pathway in the pathogenesis of CKD-PEW muscle atrophy.

### Limitations of this study

The sample size of this study is relatively small, and more samples need to be included for further study. The result of transcriptome sequencing in this study needs further biological validation which is the reseach we are currently conducting now.

## Data Availability

The datasets generated and/or analysed during the current study are available in the [GSA] repository, [https://ngdc.cncb.ac.cn/gsa-human/s/VDirO5iB].
